# Targeting ApoE4 with Nanobodies in an iPSC‐Derived Neuron Model of Alzheimer's Disease

**DOI:** 10.1002/alz70855_105524

**Published:** 2025-12-24

**Authors:** Laure Vandevelde, Olivier Zwaenepoel, Kevin Braeckmans, Jan Gettemans

**Affiliations:** ^1^ Ghent University, Ghent, Oost‐Vlaanderen, Belgium

## Abstract

**Background:**

The *APOE‐ε4* allele, encoding apolipoprotein (apo) E4—an isoform of the *APOE* gene in humans—is the strongest genetic risk factor for Alzheimer's disease (AD). However, the exact mechanisms by which it increases AD risk are still not fully understood.

Nanobodies are versatile tools to uncover new insights into the role of apoE4 and its pathological effects in AD. Anti‐apoE4 nanobodies can be employed as intrabodies to target apoE4 within induced pluripotent stem cell (iPSC)‐derived neurons, which serve as a human model system to study apoE4's role in AD pathogenesis.

**Methods:**

A llama was immunized with apoE4. The nanobodies were characterized by immunoprecipitation reactions with apoE4, apoE3, the *N*‐terminus of ApoE4 and the C‐terminus of ApoE4. Selected nanobodies were transfected together with apoE4 in HEK293T cells after which their colocalization was determined and the ability of the nanobodies to pull down apoE4 in the intracellular context. Two iPSC cell lines, homozygous for apoE4 and apoE3 respectively, were differentiated into neurons. The difference in amyloid beta 1‐42 and phospo‐tau levels between E4/E4 neurons and E3/E3 neurons will be determined (ELISA). Nanobodies will be equipped with a degradation tag (cloning) and will be delivered in the neurons via laser‐induced photoporation (recombinant nanobody or mRNA).

**Results:**

Llama immunization led to a set of apoE4 targeting nanobodies which was further reduced to a couple of nanobodies after their further characterization (specificity, affinity, apoE4 *N*‐terminus or C‐terminus binder). The co‐transfection of each nanobody and apoE4 in HEK293T cells determined if the selected nanobodies are functional as intrabodies. Differentiation of an apoE4 homozygous iPSC cell line to neurons led to a human cell model to study ApoE4's role in AD. The amyloid beta 1‐42 and phospho‐tau levels are expected to be elevated in E4/E4 neurons compared to E3/E3 neurons. Delivery of an apoE4 targeting nanobody (with a degradation tag) into apoE4 expressing neurons allows to manipulate apoE4: the protein will be degraded. The effect of this apoE4 degradation on amyloid beta 1‐42 and phospho‐tau levels will be assessed.

**Conclusion:**

Nanobodies offer a powerful tool to investigate ApoE4 within the cell.

